# Arthroscopic Rotator Cuff Repair With a Compressed Long Head of the Biceps Tendon Interposition Graft

**DOI:** 10.1002/atn2.70236

**Published:** 2026-08-27

**Authors:** Wei‐jie Gong, Han‐tao Jiang, Rang‐teng Zhu

**Affiliations:** ^1^ Department of Orthopedics Taizhou Hospital of Zhejiang Province Affiliated to Wenzhou Medical University Taizhou China

## Abstract

Incomplete healing and structural failure remain major challenges after arthroscopic rotator cuff repair, particularly in large or chronic tears. These intrinsic tissue limitations continue to restrict durable tendon‐to‐bone healing, even in the presence of advances in suture configurations and anchor technology. Consequently, biologic augmentation strategies targeting the tendon‐bone interface have gained increasing attention to enhance structural integrity and optimize the healing environment. Among available autograft options, the long head of the biceps tendon provides a biologically compatible and readily accessible tissue source that has been previously incorporated into rotator cuff reconstruction techniques. Here, we describe the use of a compressed autograft long head of the biceps tendon graft as an interposition graft in double‐row rotator cuff repair.

## Graphical abstract

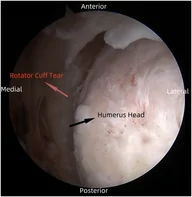

**VIDEO 1:** With the patient in the lateral decubitus position, diagnostic arthroscopy of the right shoulder was performed, and the subacromial space was inspected through a posterolateral portal. **Supra‐pectoral biceps harvest (00 min 00 s‐00 min 33 s):** The long head of the biceps tendon (LHBT) was identified in the supra‐pectoral interval using a prior anchor suture for localization. After transection, tenodesis was performed, and the proximal tendon was retrieved through the lateral portal for measurement and autograft preparation. **Subacromial preparation and medial‐row fixation (00 min 33 s‐00 min 57 s):** The arthroscope is introduced into the subacromial space for bursectomy, with acromioplasty performed as indicated. The rotator cuff tear is mobilized, the footprint is prepared, and medial‐row anchors are placed with sutures passed through the tendon for later fixation. **Graft preparation and compression (00 min 57 s‐01 min 12 s):** The harvested LHBT was compressed between plates in a graft press for 3 minutes to create a flat, uniform patch. Upon release of the compression plates, a newly created biceps tendon patch was obtained. **Biceps patch creation and suture loading (01 min 12 s‐01 min 21 s):** Upon release of the compression plates, a flattened LHBT patch is obtained. Controlled compression flattens tendon transforms the cylindrical tendon into a uniform, planar graft, thereby increasing the tendon‐bone contact surface area and facilitating interpositional placement at the tendon‐bone interface. Four absorbable sutures are placed at the graft corners to create a stable quadrilateral, with gentle traction applied to maintain uniform tension and prevent folding during insertion. **Patch delivery and fixation and dynamic arthroscopic assessment (01 min 21 s‐03 min 48 s):** An arthroscopic cannula is introduced through the lateral portal to establish a working channel. The prepared LHBT graft is oriented extracorporeally with the sutured surface facing the rotator cuff. The medial‐row sutures are first passed through the rotator cuff tendon and then sequentially passed through the graft to establish graft‐tendon integration. To facilitate insertion, the medial edge of the graft is gently folded to reduce bulk. Using a synchronized push‐pull technique, the graft is introduced between the residual supraspinatus tendon and the prepared footprint. Dynamic arthroscopy confirms smooth graft motion without impingement and stable augmentation of the repaired rotator cuff with a well‐incorporated autologous biceps patch. Video content can be viewed at https://doi.org/10.1002/atn2.70236.

Rotator cuff tears (RCTs) are a common cause of shoulder pain and functional limitation.^1^ Despite advancements in surgical techniques, failure rates remain substantial, particularly in large, chronic, or degenerative tears.^2^ Multiple factors contribute to repair failure, such as patient age, tear size, fatty infiltration, muscle atrophy, and systemic comorbidities.^3^, ^4^, ^5^ Among these, inadequate tendon‐to‐bone healing is recognized as a critical factor limiting long‐term repair integrity.^6^, ^7^

In large or retracted rotator cuff repair (RCR), direct tendon‐to‐bone repair is frequently limited by poor tissue quality and excessive repair tension, even after adequate mobilization. To address these challenges, graft‐based strategies have been increasingly explored to enhance structural support and improve the biologic environment at the tendon‐bone interface.^8^ Interpositional graft techniques involve placement of an autograft or allograft between the residual cuff tissue and the prepared footprint to augment repair stability.^8^ Within this repair strategy, the long head of the biceps tendon (LHBT) has gained attention because of its biologic compatibility, availability, and cost‐effectiveness.^9^

Several techniques incorporating the LHBT into the repair construct have been described, including compressed LHBT patch augmentation and knotless all‐suture anchor‐based techniques. Although promising, these approaches typically rely on modular compression systems and dedicated graft‐delivery devices, increasing procedural complexity. Here, we describe a simplified arthroscopic technique using a compressed autologous LHBT patch as an interposition graft in chronic, massive RCT.

## SURGICAL TECHNIQUE

### Step 1: Preoperative Workup

Preoperative evaluation is performed to confirm rotator cuff pathology and identify candidates for augmentation. All patients scheduled for arthroscopic RCR undergo physical examination, radiographs, and magnetic resonance imaging. Surgery is considered for those who fail appropriate nonoperative management.^10^, ^11^ Biologic augmentation is typically considered for large or massive tears (≥2 tendons or >2 cm retraction).^12^, ^13^ Additional risk factors such as advanced age, chronicity, revision surgery, poor tendon quality, and fatty infiltration are considered when planning augmentation.^3^, ^5^, ^14^ Patients meeting these criteria are counseled preoperatively regarding patch augmentation.

### Step 2: Surgical Positioning and Diagnostic Arthroscopy

The procedure is performed in the lateral decubitus position under general anesthesia with interscalene block. A 30° arthroscope (Arthrex, Naples, FL) is used. Standard anterior, anterolateral, lateral, and posterior portals are established, with the posterior portal serving as the primary viewing portal. Diagnostic arthroscopy is performed to assess rotator cuff pathology. When pathology of the LHBT is identified (e.g., partial tear, subluxation, or dislocation in the bicipital groove), the tendon is released from its superior labral origin using an arthroscopic biter.

### Step 3: Biceps Tenodesis

After arthroscopic detachment of the LHBT from the superior glenoid, a supra‐pectoral biceps tenodesis is performed while preserving sufficient tendon length for subsequent graft preparation. With the arthroscope in the posterior portal and instruments through the anterior portal, soft tissue over the bicipital groove is debrided to expose the LHBT beneath the transverse humeral ligament. The transverse humeral ligament is released to mobilize the tendon, and the supra‐pectoral tenodesis site just proximal to the superior border of the pectoralis major is prepared by burr decortication (Arthrex, Naples, FL) until punctate bleeding is observed. One 4.5‐mm knotless anchor (Ligatech Medical Devices, Shanghai, China) is then inserted under a drill guide. Whipstitch sutures are passed through the tendon, loaded into the anchor eyelet, and tensioned to secure the tendon. After completion of tenodesis fixation, the tendon is transected at the superior border of the transverse humeral ligament. The proximal redundant LHBT segment is then retrieved through the anterior portal for use as the autograft for compression and patch augmentation.

### Step 4: Subacromial Preparation

**FIGURE 1:**
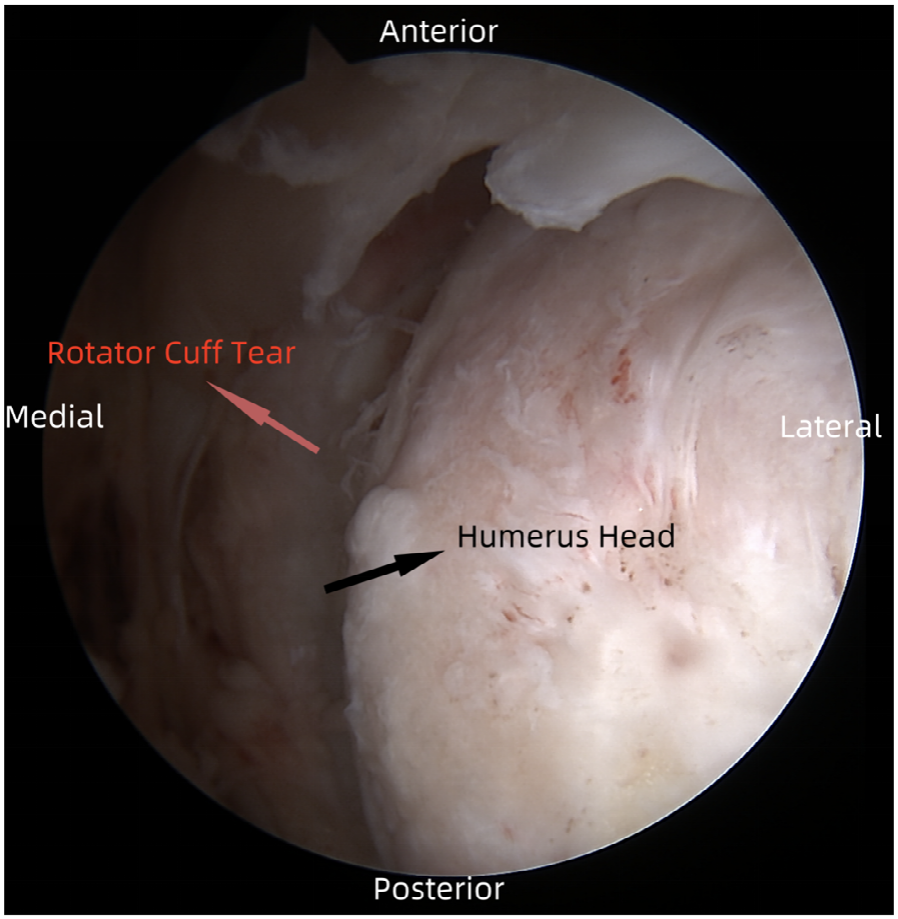
Arthroscopic view of the right shoulder in the lateral decubitus position, visualized from the posterior portal in the subacromial space. A large supraspinatus tear is identified (red arrow), exposing the humeral head (black arrow).

The arthroscope is introduced into the subacromial space through the posterolateral portal. Under spinal‐needle guidance, a lateral working portal is created at the midpoint of the lateral acromial border using an outside‐in technique. Complete bursectomy is performed to optimize visualization. When indicated, selective coracoacromial ligament release and acromioplasty are undertaken. The torn rotator cuff is mobilized to optimize reduction and minimize repair tension (Figure 1). If full anatomic reduction cannot be achieved without excessive tension, partial repair with placement of an interposition graft is performed. The greater tuberosity is decorticated to create a bleeding surface. Two 4.5‐5.5 mm medial‐row knotless anchors (Ligatech Medical Devices, Shanghai, China) are placed adjacent to the articular margin. Sutures are passed through the tendon in a looped or independent horizontal mattress configuration according to tissue quality and tear pattern, and the suture limbs are parked for subsequent lateral‐row fixation.

### Step 5: Repair Augmentation

#### Graft Preparation

**FIGURE 2:**
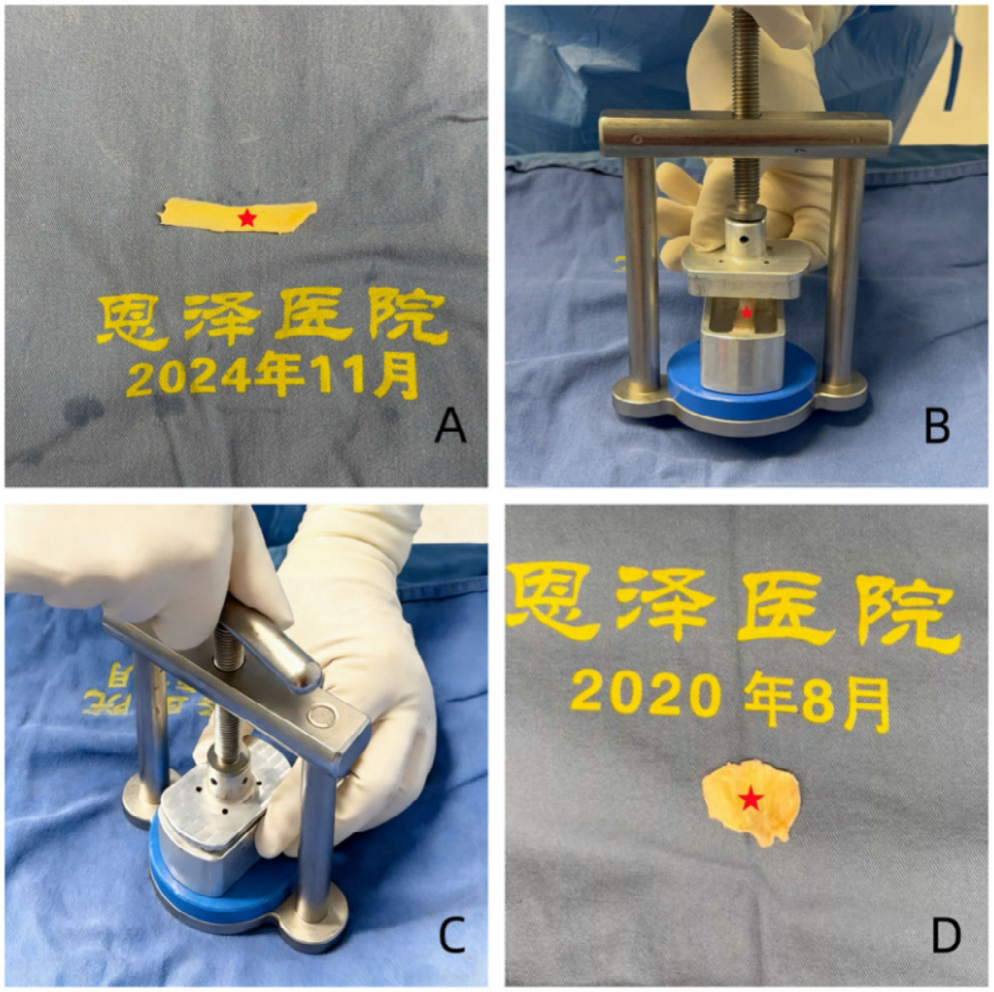
Step‐by‐step extracorporeal preparation and compression of the autologous long head of the biceps tendon (LHBT) graft. (A) The harvested LHBT (asterisk) is shown at its original length prior to preparation. (B) The LHBT graft (asterisk) is positioned between two flat compression plates of a custom tendon press device. (C) The compression device is manually tightened and maintained under compression for 3 min. (D) Final appearance of the compressed LHBT autograft (asterisk), showing a flattened graft configuration suitable for interpositional placement.

After supra‐pectoral LHBT tenodesis, the redundant proximal stump is retrieved through the lateral portal and compressed between two custom‐made flat plates (3 × 5 cm) using a threaded compression screw for 3 to 5 min, forming a flat patch measuring approximately 2 × 3 cm (Figure 2). The graft is kept continuously hydrated with sterile saline during compression. Four absorbable sutures are placed in a quadrilateral configuration. The proximal sutures are passed through the rotator cuff and retrieved externally to guide graft delivery, whereas the distal sutures remain externalized to provide traction and maintain graft orientation.

#### Delivery and Integration with the Repair

**FIGURE 3:**
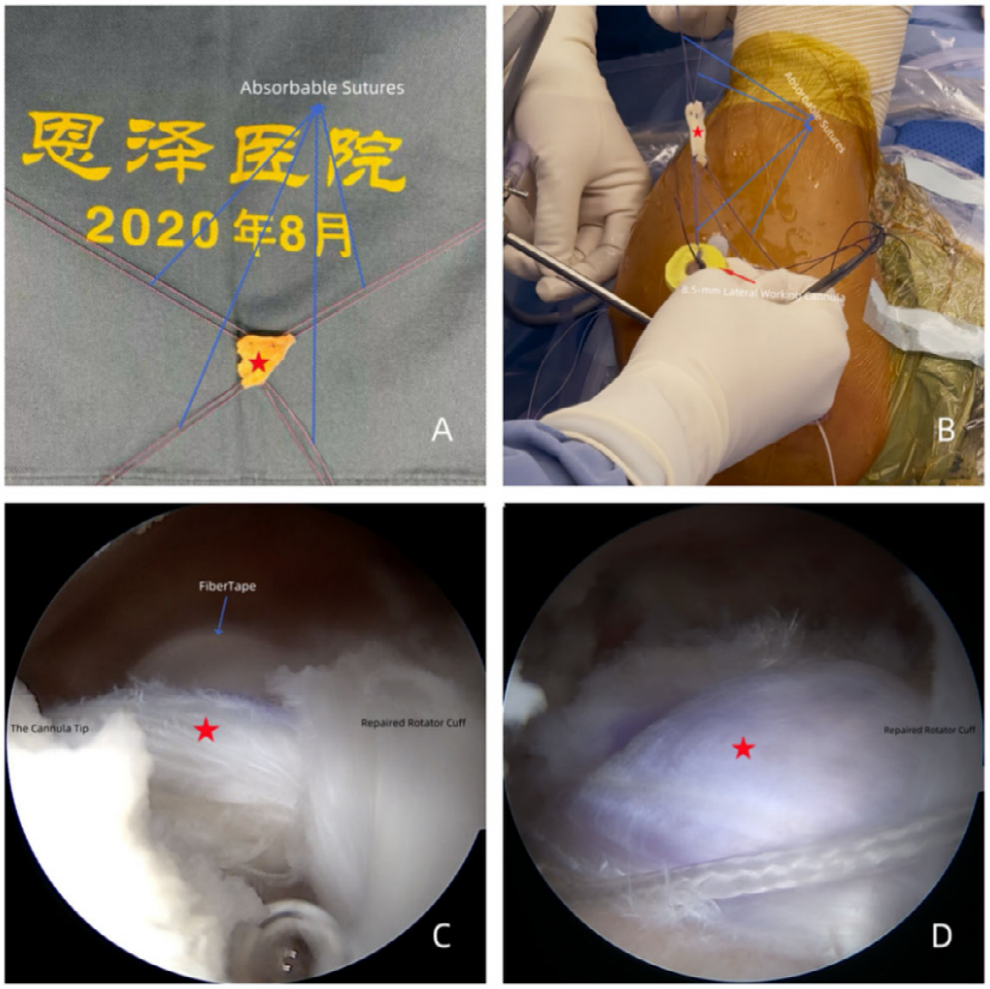
Step‐by‐step arthroscopic delivery and fixation of the long head of the biceps tendon (LHBT) autograft in a right shoulder positioned in the lateral decubitus position. (A) Four absorbable sutures (blue arrow) are passed extracorporeally through the LHBT graft (asterisk) in preparation for graft delivery. (B) External view of the right shoulder in the lateral decubitus position. Traction sutures from the LHBT autograft (asterisk) are retrieved through an 8.5‐mm lateral working cannula in preparation for arthroscopic delivery into the subacromial space. (C) Arthroscopic view of the right shoulder from the posterolateral portal in the subacromial space. The graft (asterisk) is delivered between the residual supraspinatus tendon and the prepared greater tuberosity footprint using a synchronized “push‐pull” technique through the lateral working cannula and is carefully unfolded under direct visualization. FiberTape (blue arrow) are incorporated into the repair construct. (D) Arthroscopic view of the right shoulder in the lateral decubitus position from the lateral portal. The LHBT autograft (asterisk) is positioned between the residual supraspinatus tendon and the prepared greater tuberosity footprint, showing the final interposition graft construct within a knotless double‐row repair.

**FIGURE 4:**
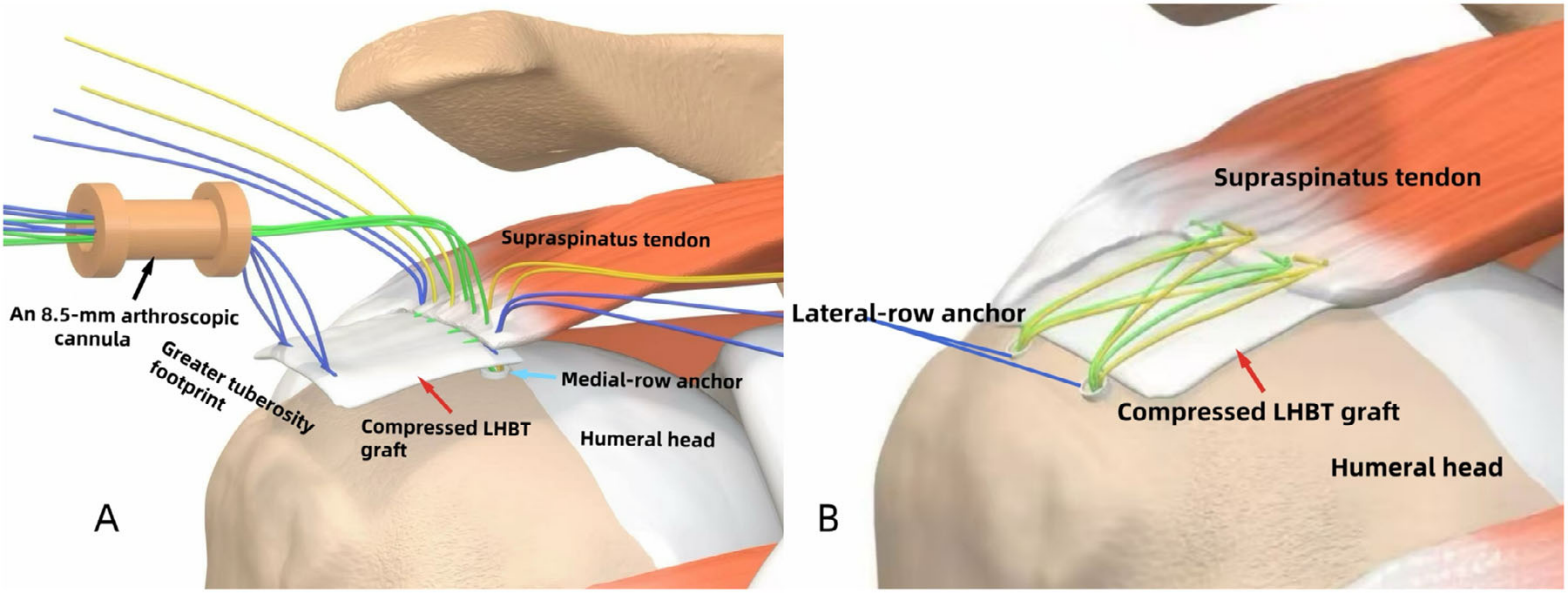
Schematic illustration of partial rotator cuff repair with an interposition graft in a right shoulder in the lateral decubitus position. (A) Coronal view showing placement of the compressed long head of the biceps tendon autograft between the residual supraspinatus tendon and the prepared greater tuberosity footprint. Medial‐row anchors are placed adjacent to the articular margin, and medial‐row sutures (green and yellow) pass through both the rotator cuff tendon and the graft to integrate the graft into the repair construct. Blue sutures represent traction sutures used during graft delivery. A lateral 8.5‐mm arthroscopic cannula is shown. (B) Coronal view of the final construct after fixation. Medial‐row sutures (green and yellow) traverse the rotator cuff tendon and graft, and lateral‐row knotless anchors secure the suture limbs laterally, compressing the graft against the greater tuberosity footprint to complete the knotless double‐row repair configuration. (LHBT, long head of the biceps tendon.)

All sutures are managed through a standard 8.5‐mm arthroscopic cannula (Arthrex, Naples, FL) in a consistent sequence to prevent entanglement. The internal sealing diaphragm of the cannula is removed to reduce resistance during insertion. Before insertion, the graft is oriented extracorporeally with the sutured surface facing the rotator cuff. After the proximal sutures are passed through the cuff, the medial edge of the graft is gently folded and introduced between the residual cuff tissue and the prepared footprint using a synchronized push‐pull technique (Figure 3A‐C). The graft is advanced under arthroscopic visualization while traction sutures are tensioned for controlled deployment. The traction sutures are used solely for graft delivery and are removed once satisfactory positioning is achieved. The medial‐row FiberTape (Arthrex, Naples, FL) limbs are then secured into two 5.5‐mm lateral‐row knotless anchors (Ligatech Medical Devices, Shanghai, China) placed along the lateral aspect of the greater tuberosity. Tension is applied to compress the graft at the tendon‐bone interface, completing the knotless double‐row construct (Video 1, Figure 3D). A schematic illustration of graft placement and suture configuration is provided in Figure 4.

### Step 6: Rehabilitation Protocol

All patients follow a standardized rehabilitation protocol. Immobilization in an abduction sling is maintained for 6 weeks, with active motion and resisted biceps activity prohibited. From 6 to 12 weeks, active‐assisted and active range of motion exercises are initiated while avoiding resisted biceps loading. After 12 weeks, rotator cuff and scapular stabilizer strengthening begin, followed by resisted biceps exercises. Return to unrestricted activities is permitted at 5 to 6 months based on clinical and imaging evaluation. Pearls and pitfalls are summarized in Table 1, and advantages and disadvantages are listed in Table 2.

**TABLE 1:** Pearls and Pitfalls

Pearls
The surgeon preserves sufficient LHBT length during supra‐pectoral tenodesis.
Adequate tendon length allows proper graft compression and patch preparation.
The surgeon maintains continuous hydration of the LHBT during compression.
Continuous hydration prevents graft desiccation and tissue damage.
The surgeon places 4 sutures at the graft corners in a quadrilateral configuration.
The quadrilateral suture configuration facilitates controlled graft delivery and orientation.
The surgeon orients the graft extracorporeally before insertion.
Proper graft orientation prevents suture crossing and graft malrotation.
The surgeon uses a synchronized push‐pull technique during graft insertion.
The push‐pull technique enables smooth interposition graft deployment.
The surgeon applies gradual tension during lateral‐row fixation.

Pitfalls
Inadequate LHBT quality compromises graft integrity and fixation strength.
Incorrect graft orientation causes suture entanglement.
Advanced glenohumeral arthritis limits the indication for this technique.

LHBT, long head of the biceps tendon.

**TABLE 2:** Advantages and Disadvantages

Advantages
This technique uses an autologous long head of the biceps tendon graft.
The autologous graft avoids immunogenic reactions.
The technique avoids donor‐site morbidity associated with allograft tissue.
The technique simplifies graft preparation by using a basic compression method.
The method eliminates the need for modular compression systems.
The approach avoids specialized graft delivery devices.
The technique integrates seamlessly with double‐row rotator cuff repair constructs.

Disadvantages
This technique requires sufficient LHBT quality.
Additional procedure with prolonged operative time and learning curve.
The thickness and quality of the compressed graft may vary depending on operator experience.

LHBT, long head of the biceps tendon.

## DISCUSSION

The management of large or massive RCT remains a significant challenge for both surgeons and patients.^12^ Numerous strategies have been proposed to reduce failure rates in the treatment of large or massive RCT, including graft‐based augmentation techniques. Currently, treatment options range from reverse total shoulder arthroplasty and superior capsular reconstruction to tendon transfers, subacromial balloon spacers, biological tuberoplasty, and partial RCR with or without augmentation.^15^, ^16^ Strategies aimed at preserving native joint anatomy and restoring functional continuity have gained increasing attention. Interpositional graft techniques represent one such approach, with multiple graft options have been described, including human dermal allograft, fascia lata autograft, and tenotomized biceps autograft.^8^

Preclinical and clinical studies have shown that interposition grafts may improve the biomechanical stability of the repair construct.^17^ In a comparative study with a minimum follow‐up of 7 years, Mori et al. showed that partial RCR augmented with a fascia lata autograft was associated with higher Constant scores and greater Constant strength compared with partial repair alone.^18^ A recent clinical study by San Pastor et al. showed improved tendon healing and functional outcomes with LHBT augmentation compared with repair alone.^19^ Collectively, these findings support the rationale for graft‐based augmentation strategies in large RCT and underscore the importance of graft selection. Among available autologous options, the LHBT represents a readily available and biologically compatible graft source.

Our technique integrates partial repair with placement of an interposition graft at the tendon‐bone interface within a knotless double‐row construct. Unlike classic bridging reconstruction for completely irreparable tears, this approach preserves residual cuff tissue and incorporates it into the repair while enhancing structural continuity. Compared with previously described techniques that rely on modular compression systems or specialized graft‐delivery devices,^20^, ^21^ the present method simplifies graft preparation using of a basic compression technique. Graft delivery is performed through a standard arthroscopic cannula without the need for specialized graft spreaders or delivery devices, potentially reducing instrumentation requirements and procedural complexity.

Nevertheless, this technique has limitations. First, this report describes a technical approach without comparative clinical or biomechanical data, and its long‐term outcomes remain to be determined. Second, the technique may not be suitable for all large RCT, particularly in cases with poor biceps tendon quality, severe tendon degeneration, or advanced glenohumeral arthritis.

## DISCLOSURES

The authors (W.G., H.J., R.Z.) declare that they have no known competing financial interests or personal relationships that could have appeared to influence the work reported in this article.
